# Unveiling Key Biomarkers and Therapeutic Drugs in Polycystic Ovary Syndrome (PCOS) Through Pathway Enrichment Analysis and Hub Gene-miRNA Networks

**DOI:** 10.5812/ijpr-139985

**Published:** 2023-11-20

**Authors:** Roozbeh Heidarzadehpilehrood, Maryam Pirhoushiaran, Malina Binti Osman, King-Hwa Ling, Habibah Abdul Hamid

**Affiliations:** 1 Department of Obstetrics & Gynaecology, Faculty of Medicine and Health Sciences, Universiti Putra Malaysia, 43400, Serdang, Selangor, Malaysia; 2 Department of Medical Genetics, School of Medicine, Tehran University of Medical Sciences, 1417613151, Tehran, Iran; 3 Department of Medical Microbiology, Faculty of Medicine and Health Sciences, Universiti Putra Malaysia, 43400, Serdang, Selangor, Malaysia; 4 Department of Biomedical Science, Faculty of Medicine and Health Sciences, Universiti Putra Malaysia, 43400, Serdang, Selangor, Malaysia; 5 Malaysian Research Institution on Ageing, (MyAgeing), Universiti Putra Malaysia, 43400, Serdang, Selangor, Malaysia

**Keywords:** Polycystic Ovary Syndrome, Bioinformatics, Biomarkers, MicroRNAs, Drug-Target Network

## Abstract

**Background:**

Polycystic ovary syndrome (PCOS) affects women of reproductive age globally with an incidence rate of 5% - 26%. Growing evidence reports important roles for microRNAs (miRNAs) in the pathophysiology of granulosa cells (GCs) in PCOS.

**Objectives:**

The objectives of this study were to identify the top differentially expressed miRNAs (DE-miRNAs) and their corresponding targets in hub gene-miRNA networks, as well as identify novel DE-miRNAs by analyzing three distinct microarray datasets. Additionally, functional enrichment analysis was performed using bioinformatics approaches. Finally, interactions between the 5 top-ranked hub genes and drugs were investigated.

**Methods:**

Using bioinformatics approaches, three GC profiles from the gene expression omnibus (GEO), namely gene expression omnibus series (GSE)-34526, GSE114419, and GSE137684, were analyzed. Targets of the top DE-miRNAs were predicted using the multiMiR R package, and only miRNAs with validated results were retrieved. Genes that were common between the “DE-miRNA prediction results” and the “existing tissue DE-mRNAs” were designated as differentially expressed genes (DEGs). Gene ontology (GO) and pathway enrichment analyses were implemented for DEGs. In order to identify hub genes and hub DE-miRNAs, the protein-protein interaction (PPI) network and miRNA-mRNA interaction network were constructed using Cytoscape software. The drug-gene interaction database (DGIdb) database was utilized to identify interactions between the top-ranked hub genes and drugs.

**Results:**

Out of the top 20 DE-miRNAs that were retrieved from the GSE114419 and GSE34526 microarray datasets, only 13 of them had “validated results” through the multiMiR prediction method. Among the 13 DE-miRNAs investigated, only 5, namely *hsa-miR-8085*, *hsa-miR-548w*, *hsa-miR-612*, *hsa-miR-1470*, and *hsa-miR-644a*, demonstrated interactions with the 10 hub genes in the hub gene-miRNA networks in our study. Except for *hsa-miR-612*, the other 4 DE-miRNAs, including *hsa-miR-8085*, *hsa-miR-548w*, *hsa-miR-1470*, and *hsa-miR-644a*, are novel and had not been reported in PCOS pathogenesis before. Also, GO and pathway enrichment analyses identified “pathogenic *E. coli* infection” in the Kyoto encyclopedia of genes and genomes (KEGG) and “regulation of Rac1 activity” in FunRich as the top pathways. The drug-hub gene interaction network identified *ACTB*, *JUN*, *PTEN*, *KRAS*, and *MAPK1* as potential targets to treat PCOS with therapeutic drugs.

**Conclusions:**

The findings from this study might assist researchers in uncovering new biomarkers and potential therapeutic drug targets in PCOS treatment.

## 1. Background

Polycystic ovary syndrome (PCOS), a prevalent endocrine and metabolic condition among women of reproductive age, is estimated to affect 5% to 26% of women worldwide, with variability depending on the diagnostic criteria utilized (1). The clinical necessity for robust and precise genetic biomarkers, both in tissue and blood specimens, has intensified. These biomarkers hold the promise of unraveling the complex molecular mechanism of PCOS and enhancing diagnostic accuracy (2, 3), hence providing important support for clinical translational research initiatives (4, 5). Noncoding RNAs are being recognized as crucial regulators of gene expression, organizing complex molecular networks that are essential to the pathophysiology of numerous diseases (6-9). Translational studies have investigated the complex systems biology of diseases, particularly PCOS, using various bioinformatics approaches, microarray data analysis, DNA and RNA next-generation sequencing methods, and DNA methylation analysis, among others (10-12).

Granulosa cells (GCs) encompass the oocyte, coordinating follicular development and playing a pivotal role in primordial follicle maturation. Beyond their central role in normal folliculogenesis, GCs contribute to the perturbed follicular dynamics characteristic of conditions like PCOS (13). Ovaries of individuals with PCOS manifest small antral follicles, which exhibit impaired progression into dominant follicles. Notably, increased GC proliferation within smaller follicles is evident in PCOS-affected ovaries (14). Consequently, GCs present a valuable cellular model for investigating the intricacies of PCOS.

MicroRNAs (miRNAs), a class of small noncoding RNAs, exert a substantial regulatory effect on gene expression. Within GCs, a variety of microRNAs coordinate ovarian follicle development and function (15) by directly interacting with specific molecular targets and modulating diverse signaling pathways. These pathways involve important processes such as atresia, ovulation, and ovarian steroidogenesis, with key contributors including *TGF-β1* (16), *FSH* (17), hormones (18, 19), and apoptosis-related pathways (20). In our search, we analyzed three distinct gene expression omnibus series (GSE) datasets derived from PCOS patients: GSE34526, GSE114419, and GSE137684. Our primary objective was to identify top DE-miRNAs with validated effects, utilizing the predictive power of the multiMiR R package. Genes that were common between the “DE-miRNA prediction results” and the “existing tissue DE-mRNAs” were designated as differentially expressed genes (DEGs). Then, DEGs were exposed to functional enrichment analysis, followed by the construction of hub miRNA-gene networks. Finally, the drug-gene interaction database (DGIdb) was used to investigate the interactions between the initial top 5 ranked hub genes and relevant therapeutic drugs.

## 2. Objectives

The objectives of this study were to identify the top differentially expressed miRNAs (DE-miRNAs) and their corresponding targets in hub gene-miRNA networks, as well as identify novel DE-miRNAs by analyzing three distinct microarray datasets. Additionally, functional enrichment analysis was performed using bioinformatics approaches. Finally, interactions between the 5 top-ranked hub genes and drugs were investigated.

## 3. Methods

### 3.1. Downloading Microarray Data

The miRNA and mRNA expression profiles of GSE114419 (accessed October 30, 2018) and GSE34526 (accessed November 6, 2012) and the mRNA expression profile of GSE137684 (accessed September 19, 2019) were downloaded from the gene expression omnibus (GEO) database.

### 3.2. Statistical Analysis and Preprocessing of Microarray Data for Integration

R project version 4.0.1 and the annotation package were used to process the downloaded matrix file and platform. The gene symbol, or the international standard name of the gene, was generated from the ID, matching the probe name. An inter-array normalization was performed using the normalize between arrays function from the limma R package. Top miRNAs and mRNAs from three microarray datasets were analyzed for differential expression analysis in the GCs of PCOS and healthy participants using the R software package limma. The cutoff parameters for screening top DE-miRNAs were as follows: P-value < 0.05, log2FC ≥ 0.80, and log2FC ≤ -0.50.

### 3.3. Identification of Top DE-miRNAs and DE-mRNAs in Three Microarray Datasets

The microarray dataset GSE137684 exclusively contains mRNA expression data, whereas both GSE114419 and GSE34526 encompass both miRNA and mRNA expression patterns. Regarding this issue and based on the cutoff parameters detailed in section 3.2, top DE-miRNAs were retrieved from GSE114419 and GSE34526 microarray datasets. The predicted targets of these top DE-miRNAs were retrieved using the multiMiR R package. The "multiMiR" R package includes various functions, one of which is for "identifying validated miRNA targets". The validated miRNA targets are those targets in this package that have been demonstrated to be accurate by means of proven experimental procedures, including RT-PCR, Western blot, luciferase reporter assay, in situ hybridization, HITS-CLIP, degradome sequencing, and other methods. Also, among the databases in the multiMiR package, only the “validated results” of two databases, including miRTarBase and TarBase, were used for analysis.

All of the microarray datasets mentioned in the manuscript, including GSE137684, GSE114419, and GSE34526, encompass mRNA expression profiles. Regarding this issue and based on the cutoff parameters detailed in section 3.2, top DE-mRNAs among these three microarray datasets were retrieved.

### 3.4. Identification of Differentially Expressed Genes

In our study, DEGs are defined as genes overlapping between “validated multiMiR prediction results of the top 13 DE-miRNAs” and “existing tissue DE-mRNAs”. A Venn diagram was created using an online resource to visually display the genes that overlap between the identified sets.

### 3.5. Gene Ontology Enrichment Analysis of Differentially Expressed Genes

Gene ontology (GO) enrichment analysis, including biological processes (BPs), molecular functions (MFs), and cellular components (CCs), was performed using ShinyGO v0.77 (accessed April 19, 2022) with an FDR cutoff of 0.05 (21). The Kyoto encyclopedia of genes and genomes (KEGG) pathway enrichment analysis on DEGs was built using R packages including DOSE, Hs.egENSEMBL, and clusterProfiler: DOSE R package for disease ontology semantic and enrichment analysis, Hs.egENSEMBL R package for mappings between entrez gene identifiers and KEGG pathway, and clusterProfiler for comparing biological themes among gene clusters. Also, the open-source and user-friendly software FunRich was used for the biological pathway analysis of DEGs.

### 3.6. Construction of Protein-Protein Interaction and Hub Gene-miRNA Networks

First, protein-protein interactions (PPIs) for DEGs were created using the STRING v11.5 database (accessed August 12, 2021), an online tool (22), and a list of 352 mRNAs uploaded to the STRING database. In the next step, the Cytoscape v3.7.2 software plugin, CytoHubba, was used to assess and investigate significant nodes inside biological networks, using diverse network properties for scoring purposes. The CytoHubba plugin conveniently offers 11 distinct topological analysis approaches in a single package. Hence, for scoring and identifying significant nodes in our PPI network, we used the CytoHubba plugin. Using this plugin, 10 genes were defined as hub genes. Additionally, the CytoHubba plugin was used to discover the interactions between these 10 hub genes and miRNAs to produce the hub gene-miRNA interaction network.

### 3.7. Drug-Gene Interaction Analysis

Drugs were chosen based on the initial top 5 ranked hub genes, which emerged as promising centers of interest, utilizing the (DGIdb; version 3.0.2; sha1 ec916b2). This study only included FDA-approved drugs. The DGIdb compiles information on drug-gene interactions originating from diverse sources, including the Drug Bank, ChEMBL, NCBI Entrez, Ensembl, PharmGKB, PubChem, clinical trials, and literature indexed in PubMed. Researchers may use this resource to hypothesize about therapeutic gene targets and drug development priorities (23). Interaction between drugs and genes was performed using Cytoscape (version 3.7.2).

## 4. Results

### 4.1. Identification of Top DE-mRNAs and DE-miRNAs in Three Microarray Datasets

**Figure 1.**
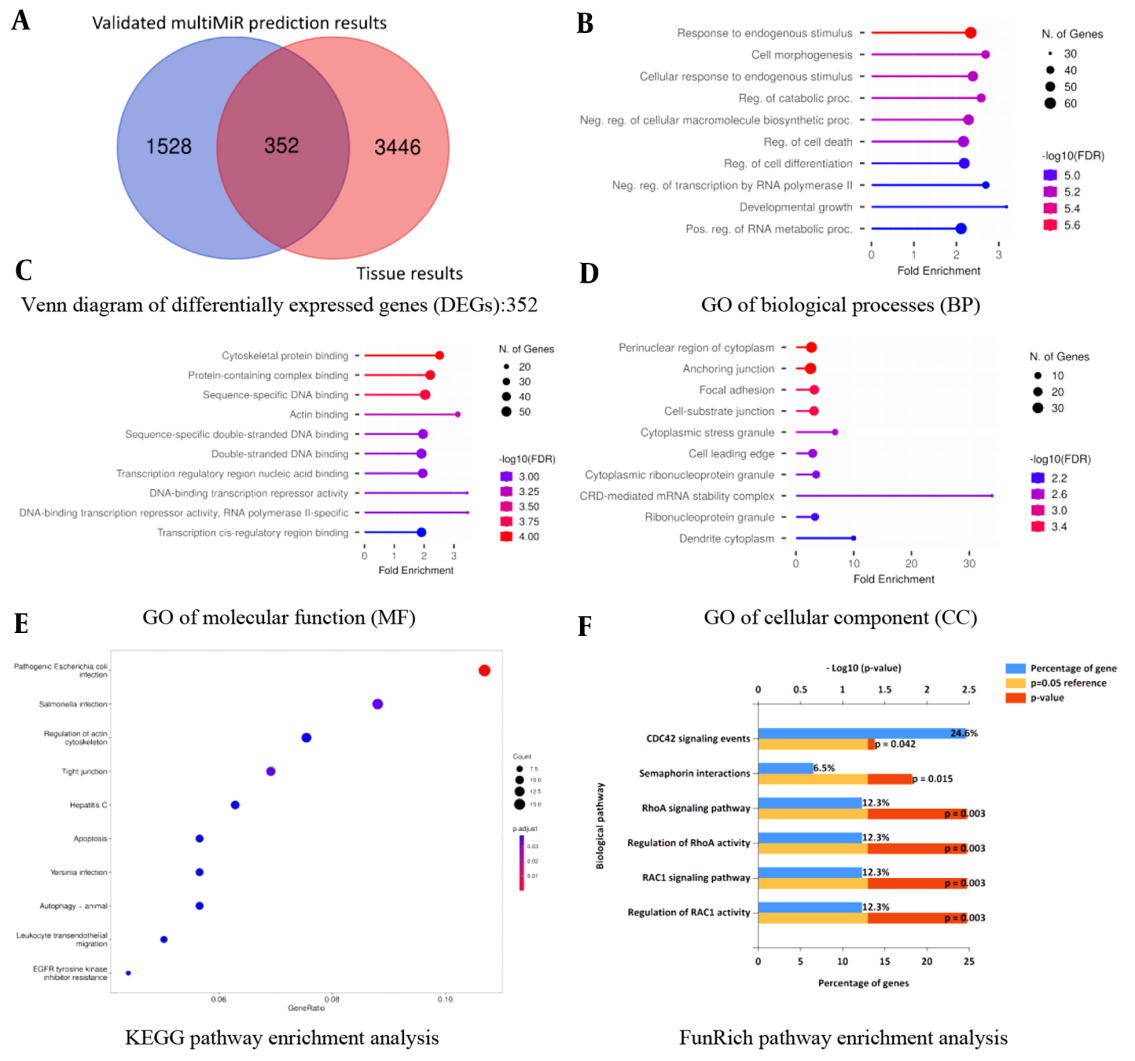
Venn diagram and gene ontology (GO) enrichment analysis of differentially expressed genes (DEGs): A, Venn diagram of 352 DEGs from three microarray datasets: DEGs are defined as genes that are common between the “validated multiMiR prediction results of the top 13 differentially expressed (DE)-miRNAs” and “existing tissue DE-miRNAs”; B, GO of biological processes (BP) enrichment analysis; C, GO of molecular function (MF) enrichment analysis; D, GO of cellular component (CC) enrichment analysis; E, Kyoto encyclopedia of genes and genomes (KEGG) pathway enrichment analysis; F, FunRich pathway enrichment analysis.

While GSE137684 exclusively presents mRNA expression data, GSE114419 and GSE34526 both include miRNA and mRNA expression patterns. Regarding this issue and based on the cutoff parameters detailed in section 3.2, the top 20 DE-miRNAs were retrieved from GSE114419 and GSE34526 microarray datasets. Out of the top 20 DE-miRNAs, only 13 of them had “validated results” through the multiMiR prediction method. These 13 DE-miRNAs consist of 10 upregulated and 3 down-regulated miRNAs (Table 1). Using only “validated results” in the multiMiR R package, “1880” miRNA targets were generated (Figure 1A, the left segment of the Venn diagram: 1528 + 352 = “1880”).

**Table 1.** Top 13 Significantly Up- and Down-Regulated miRNAs with Validated Results Through Multimir Prediction Method

miRNAs	Status	LogFC	P-Value
***hsa-miR-8085***	Up	3.32026	0.00465
***hsa-miR-612***	Up	2.924954	0.012351
***hsa-miR-637***	Up	2.890822	0.004339
***hsa-miR-1282***	Up	2.388948	0.031552
***hsa-miR-8085***	Up	2.373889	0.010707
***hsa-miR-4656***	Up	2.194869	0.026918
***hsa-miR-548w***	Up	1.338450222	0.007760057
***hsa-miR-4451***	Up	0.880255278	0.016179284
***hsa-miR-1264***	Up	0.862366056	0.001420294
***hsa-miR-644a***	Up	0.805058333	0.037980205
***hsa-miR-3146***	Down	-0.691118278	0.024531246
***hsa-miR-1470***	Down	-0.643911889	0.012536171
***hsa-miR-4473***	Down	-0.580333833	0.001855791

All of the microarray datasets mentioned in the manuscript, including GSE137684, GSE114419, and GSE34526, encompass mRNA expression profiles. Regarding this issue and based on the cutoff parameters detailed in section 3.2, top DE-mRNAs among these three microarray datasets were retrieved, which were “3798” mRNAs (Figure 1A, the right segment of the Venn diagram: 3446 + 352 = “3798”).

### 4.2. Identification of Differentially Expressed Genes

According to the methodology, DEGs are genes overlapping between “validated multiMiR prediction results of the top 13 DE-miRNAs” and “existing tissue DE-mRNAs”. Hence, using the provided definition and referring to the Venn diagram, a total of “352” DEGs were found to be differentially expressed between PCOS subjects and healthy controls (Figure 1A, overlapping segment of Venn diagram: “352”).

### 4.3. Gene Ontology and Pathway Enrichment Analysis

A significant number of genes showed enrichment in response to endogenous stimulus, cytoskeletal protein binding, and perinuclear region of cytoplasm for BP, MF, and CC, respectively. *Escherichia coli* (*E. coli*) infection and *RAC1* activity are the most significant pathways in KEGG and FunRich pathway analyses, respectively (Figure 1B-F and Table 2). 

**Table 2.** Gene Ontology Enrichment Analysis with Polycystic Ovary Syndrome

Enrichment FDR	No. of Genes	Pathway Genes	Fold Enrichment	Gene Ontology
**Biological process**				
1.66E-06	61	1769	2.339491	Response to endogenous stimulus
5.94E-06	43	1085	2.688797	Cell morphogenesis
5.94E-06	53	1505	2.389234	Cellular response to endogenous stimulus
6.20E-06	45	1181	2.585128	Reg. of catabolic proc.
6.26E-06	55	1631	2.287853	Neg. reg. of cellular macromolecule biosynthetic proc.
7.28E-06	59	1847	2.167227	Reg. of cell death
1.09E-05	56	1738	2.186038	Reg. of cell differentiation
1.1E-05	38	957	2.7	Neg. reg. of transcription by RNA polymerase II
1.1E-05	30	640	3.2	Developmental growth
1.1E-05	59	1895	2.1	Pos. reg. of RNA metabolic proc.
**Molecular function**				
9.23E-05	39	1050	2.519966	Cytoskeletal protein binding
0.000110925	47	1446	2.205205	Protein-containing complex binding
0.000145606	53	1767	2.034973	Sequence-specific DNA binding
0.00052994	22	477	3.12913	Actin binding
0.000873657	48	1660	1.96179	Sequence-specific double-stranded DNA binding
0.000931633	50	1775	1.911133	Double-stranded DNA binding
0.000955304	17	333	3.463571	DNA-binding transcription repressor activity, RNA polymerase II-specific
0.000955304	17	335	3.442893	DNA-binding transcription repressor activity
0.000955304	46	1598	1.952992	Transcription regulatory region nucleic acid binding
0.001506085	45	1596	1.91293	Transcription cis-regulatory region binding
**Cellular component**				
0.0001826	31	783	2.686082	Perinuclear region of cytoplasm
0.0001826	34	907	2.543261	Anchoring junction
0.0003813	22	471	3.168992	Focal adhesion
0.0003813	22	478	3.122584	Cell-substrate junction
0.0025373	8	80	6.784524	Cytoplasmic stress granule
0.0034097	19	445	2.896763	Cell leading edge
0.0041227	3	6	33.92262	CRD-mediated mRNA stability complex
0.0041227	14	272	3.492034	Cytoplasmic ribonucleoprotein granule
0.0062328	14	288	3.298032	Ribonucleoprotein granule
0.0071017	5	34	9.977241	Dendrite cytoplasm

### 4.4. Construction of Hub miRNA-Gene Networks

**Figure 2.**
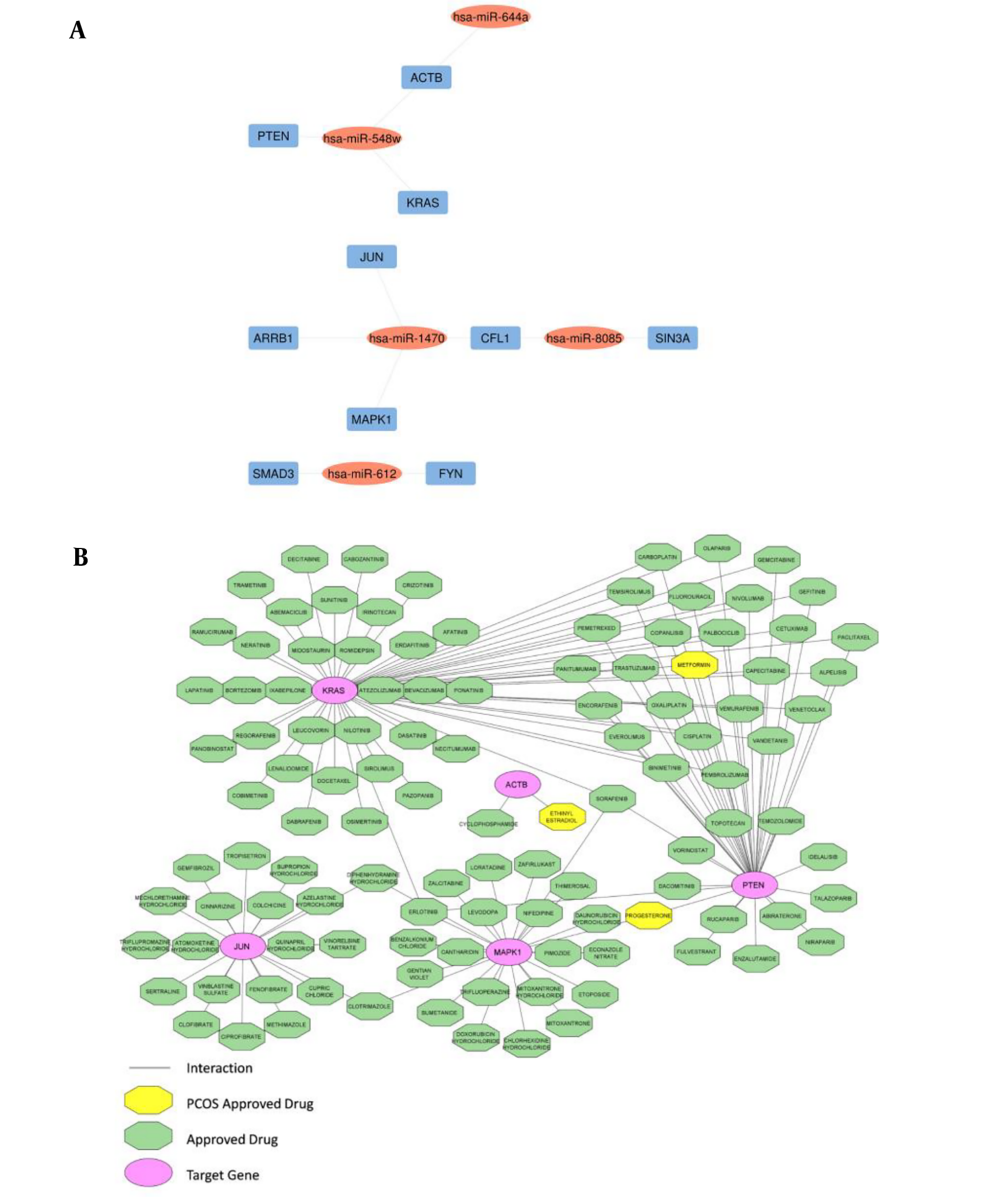
Hub gene-miRNA networks and drug-hub gene interactions; A, hub gene-miRNA networks: Blue rectangles are hub genes, including *ACTB*, *JUN*, *PTEN*, *KRAS*, *MAPK1*, *SMAD3*, *FYN*, *ARRB1*, *CFL1*, and *SIN3A* and oval red nodes are DE-miRNAs; B, drug-hub gene interactions: Oval pink nodes are the initial top 5 ranked hub genes, including *ACTB*, *JUN*, *PTEN*, *KRAS*, and *MAPK1*, which serve as potential gene targets for drugs. Green octagon nodes indicate FDA-approved drugs. Yellow octagon nodes indicate FDA-approved drugs for polycystic ovary syndrome (PCOS).

The top 10 hub genes were found through the plugin cytoHubba in the PPI network (Table 3). The network of hub DE-miRNAs and target genes was constructed through the Cytoscape (Figure 2A). 

**Table 3.** Top 10 Target Genes in Protein-Protein Interaction Network

Rank	Name	Score
**1**	*ACTB*	63
**2**	*JUN*	38
**3**	*PTEN*	36
**4**	*KRAS*	31
**5**	*MAPK1*	30
**6**	*SMAD3*	26
**7**	*FYN*	25
**8**	*ARRB1*	23
**9**	*CFL1*	22
**9**	*SIN3A*	22

### 4.5. Drug-Gene Construction Network

The DGIdb was utilized to analyze the drugs that interacted with the initial top 5 ranked hub genes, including *ACTB*, *JUN*, *PTEN*, *KRAS*, and *MAPK1*, as potential gene targets for these drugs. Through the DGIdb, 113 FDA-approved drugs were predicted as potential PCOS medications (Figure 2B). 

## 5. Discussion

Polycystic ovary syndrome is a complicated disorder that mostly affects women of reproductive age and is characterized by a variety of endocrine and metabolic abnormalities. Our study involved transcriptomic analysis of GCs in women with PCOS compared to healthy controls, followed by GO enrichment analysis, the establishment of hub miRNA-gene networks, and the construction of drug-hub gene interactions.

To the best of our knowledge, the current study is the first to reveal that DEGs involved in KEGG pathway analysis of GCs are strongly associated with *E. coli* infection. Several studies have demonstrated that PCOS is associated with alterations in the microorganisms of the intestines (24-26) and vaginal region (27-29). Nevertheless, very few studies have investigated the microorganisms in follicular fluid or GCs and their association with infertility (30). A functional experiment conducted by Pelzer et al. indicated that follicular fluid was not sterile and that it was possible to isolate bacteria from the follicular fluid of infertile women (31). Furthermore, the level of bacterial colonization of the follicular fluid was linked to fertility treatment effectiveness (29). It is currently unclear how precisely *E. coli* levels in follicular fluid affect the infertility levels in PCOS. Further functional studies should be conducted to shed light on this matter because this field represents a novel research frontier. The results of our KEGG pathway enrichment analysis demonstrated that the DEGs were mainly enriched in pathogenic *E. coli* infection, salmonella infection, and *Yersinia* infection, followed by triggering innate immune responses such as leukocyte trans-endothelial migration. According to our KEGG pathways analysis, 17 genes, including *ARF6*, *ABCF2*, *FYN*, *JUN*, *ARHGEF2*, *WIPF1*, *ACTB*, *CLDN1*, *CLDN11*, *CLDN16*, *MAPK1*, *MYO1F*, *MYO5A*, *MYH14*, *OCLN*, *PTPN6*, and *TUBB2A*, were enriched in the *E. coli* infection pathway. Most of these genes belong to TNF signaling-, NRL signaling-, cytoskeleton regulation-, and membrane trafficking pathways.

The role of the *RAC1* signaling pathway is confirmed through many reproductive events, including anchoring in oocytes (32), modulating the transcription of genes required for follicular assembly (33), and GC proliferation (34). Furthermore, Liu et al. (35) and Cozzolino and Seli (36) reported increased GC proliferation in the ovaries of PCOS in the murine models. In accordance with these findings (33-36), our study discovered that the *Rac1* signaling pathway was upregulated in the GCs of PCOS patients compared to healthy controls and was the first-ranked pathway in biological pathway analyses. According to our results, genes enriched in the *Rho* family of *GTPases* (*Rac1*, *Cdc42*, and *RhoA*) were significantly upregulated in PCOS' GCs compared to healthy controls. Because these genes are critical for cell growth and proliferation, their upregulation resulted in increased GC proliferation, probably leading to the development of PCOS (37).

In the current study, 5 miRNAs, including hsa-miR-8085, hsa-miR-548w, hsa-miR-612, hsa-miR-1470, and hsa-miR-644a, demonstrated interactions with 10 hub genes in the hub gene-miRNA networks and were defined as hub miRNAs. Except for hsa-miR-612, the other 4 DE-miRNAs, including hsa-miR-8085, hsa-miR-548w, hsa-miR-1470, and hsa-miR-644a, are novel and had not been reported in PCOS pathogenesis before. To the best of our knowledge, this is the first time that hsa-miR-8085, hsa-miR-548w, hsa-miR-1470, and hsa-miR-644a are selected as hub miRNAs associated with PCOS via multiple bioinformatics analyses in our study.

According to the findings of Peng et al., *hsa-miR-8085* is a predicted miRNA that regulates *HOXC10* expression, which plays a significant role in ovarian cancer metastasis (38). The highest expression levels of *hsa-miR-548w* were discovered in ovarian cumulus granulosa cell (CGC) and mural granulosa cell (MGC) samples according to small RNA high-throughput sequencing performed by Rooda et al. (39). In line with the results of Rooda et al. (39), upregulation of *hsa-miR-548w* was observed in our study in PCOS patients. *Has-miR-612* had an essential function in suppressing *Rap1b*, a regulator of the *MAPK* pathway, which is critical in the pathophysiology of PCOS patients with insulin resistance, according to the findings of Hu et al. (40) research. Based on our results, upregulation of *has-miR-612* plays an important role in GCs of PCOS patients, which is consistent with Hu et al. (40). Overexpression of *hsa-miR-1470* induces cell proliferation, migration, and apoptosis through different mechanisms in hepatocellular carcinoma (HCC) and esophageal squamous carcinoma cells (ESCCs). Prior to our current investigation, no association between *hsa-miR-1470* and PCOS had been reported (41, 42). As known, *has-miR-612* prevents cellular proliferation and invasion in gastric cancer, and its overexpression increases apoptosis in HCC. To the best of our knowledge, this is the first reported association between *has-miR-612* and PCOS (43, 44).

The *ACTB* gene, the top-ranked hub gene identified in our PPI construction, encodes the beta-actin protein, which is a member of the actin protein family. Shen et al. identified *ACTB* as an upregulated gene in PCOS that was positively related to actin cytoskeleton regulation and had a role in PCOS development (45). Prior research led to the concept that the Jun and Fos subfamilies, which are essential in numerous aspects of cell proliferation and differentiation, play important roles in ovarian follicular development (46). Furthermore, the activation of the ovary-specific PII promoter of the *aromatase* gene, which is responsible for the production of estrogen in premenopausal women, is functionally shown to be regulated by Jun proteins (47). These studies (46, 47) focused on the crucial functions of JUN proteins in granulosa cells, and in line with these results, we found that the JUN protein is the second most important hub gene according to our data.

In our study, the initial top 5 ranked hub genes (*ACTB*, *JUN*, *PTEN*, *KRAS*, and *MAPK1*) were identified as potential candidates for drug targets during the search for medications using the DGIdb database. According to the DGIdb database, 113 drugs with potential anti-PCOS therapeutic effectiveness were identified, the majority of which have unknown mechanisms. Currently, ethinyl estradiol (EE) serves as the estrogen in the vast majority of combined oral contraceptives (COCs). The clinical and hormonal aspects of PCOS seem to be improved by the ethinyl estradiol/DRSP combination (48). In addition, ethinyl estradiol is an effective treatment for PCOS-related hyperandrogenism-related skin symptoms (49). Ethinyl estradiol has an interaction with the *ACTB* gene in our study. Considering its interaction with ethinyl estradiol in PCOS, *ACTB* has the potential to develop into a therapeutic target for ethinyl estradiol in the near future. Overall, the progesterone level in PCOS patients is related to the clinical pregnancy rate (50). Furthermore, statistics from the pilot daily diary indicate that PCOS patients' experiences with cyclic progesterone medication have exhibited positive changes (51). In our results, progesterone interacted with *PTEN* and *MAPK1*. Results of a systematic review and meta-analysis indicated that metformin has the strongest evidence for enhancing menstrual cycles, glucose levels, and adiposity in PCOS, notably when incorporated alongside lifestyle adjustments (52, 53). Animal model research demonstrated that metformin could benefit PCOS mice with ovarian dysfunction as well as obesity and metabolic problems (54). In the present study, metformin interacted with *PTEN* and *KRAs*. As a result, these two genes may provide new therapeutic targets and are likely to open new horizons in the treatment of PCOS.

### 5.1. Conclusions

Our study on PCOS led to the identification of novel dysregulated miRNAs and pathways involved in PCOS. In the current study, multiple bioinformatics analyses were performed, which resulted in the identification of 352 DEGs and 5 hub *DE-miRNAs*, including *hsa-miR-8085*, *hsa-miR-548w*, *hsa-miR-612*, *hsa-miR-1470*, and *hsa-miR-644a*. Except for *hsa-miR-612*, the other 4 DE-miRNAs, including *hsa-miR-8085*, *hsa-miR-548w*, *hsa-miR-1470*, and *hsa-miR-644a*, are novel and had not been reported in PCOS pathogenesis before. The development of the hub gene-miRNA networks and drug-hub gene interactions might be beneficial to the investigation of the underlying causes of PCOS. The results serve as a basis for further research into the impacts of these miRNAs and their associated pathways on PCOS. Additional in vitro and in vivo research is required to validate these results.

## Data Availability

The data presented in this study were uploaded during submission through the manuscript file and are openly available for readers. Microarray data were deposited into the gene expression omnibus database under accession numbers GSE34526, GSE114419, and GSE137684.
